# Editorial: Global excellence in oral health: Europe

**DOI:** 10.3389/froh.2023.1219999

**Published:** 2023-05-31

**Authors:** V. Pavlic, L. J. Kesic

**Affiliations:** ^1^ Department of Periodontology and Oral Medicine, Medical Faculty University of Banja Luka, Banja Luka, Bosnia and Herzegovina; ^2^ The Republic of Srpska Institute of Dentistry, Banja Luka, Bosnia and Herzegovina; ^3^ Department of Periodontology and Oral Medicine, Faculty of Medicine University of Nis, Nis, Serbia

**Keywords:** oral health, promotion of oral health, world oral health day, Europe, global excellence

**Editorial on the Research Topic** Global excellence in oral health: Europe

Global collaboration is the cornerstone of scientific advancement. The aim of this Research Topic titled “Global excellence in oral health: Europe” was to highlight the latest advancements in oral health across the globe, with a specific emphasis on Europe. Our goal was to collect scientific articles from various dental area of expertise and to share knowledge, tools, and confidence on the latest research from European scientists in order to secure good oral health.

This Research Topic contains five manuscripts, including one review article and four original research articles. The first published review article by Bullock et al. summarized the medication-related osteonecrosis of the jaw (MRONJ) pathology. MRONJ is presented as necrotic bone sections exposed via lesions in the overlying soft tissue. It is a complication affecting patients who are being treated with antiresorptive or antiangiogenic medications, and most often patients receiving bisphosphonates (BPs). BPs are used to treat osteoporosis, bony metastases, and hypercalcemia of malignancy affecting bone remodeling, angiogenesis, inflammation, and soft tissue toxicity. This review article confirmed the negative effects of bisphosphonates on the viability, apoptosis, and proliferation of fibroblasts and keratinocytes, reducing the epithelial thickness and soft tissue wound healing. Bullock et al. accented the importance of better understanding the underlying cellular mechanisms of MRONJ in possible identification of targets responsible for onset and progression of MRONJ. Identification of the targets responsible for MRONJ was the aim of the second published original research article by Rattanawonsakul et al. in this Research Topic. To date, it is known that geranylgeraniol (GGOH) is a rescuer of nitrogen-containing BPs on the mevalonate pathway, seen in the mucosal breakdown of MRONJ. The authors hypothesized that GGOH could restore soft tissue impairment in MRONJ and could have therapeutic potential for BPs-induced soft tissue toxicity in MRONJ. However, the results of this study demonstrated that GGOH was itself toxic to oral mucosa cells, and therefore was not able to prevent BPs-induced soft tissue toxicity in MRONJ. Since patients with MRONJ require meticulous treatment planning and management strategies, further research is necessary to identify potential targets of MRONJ.

The third published original research article discussed the importance of understanding the negative effects of industrial noise in the morphological changes of several organs, including teeth. Industrial noise is characterized by high intensity and a wide spectrum of wavelengths that include low-frequency noise. To date, it is known that the stomatognathic system is affected by the effects of prolonged exposure to noise. Cavacas et al. wanted to realize whether industrial noise affects teeth and therefore conducted an *in vivo* study in order to evaluate changes in the pulp-dentin complex of teeth exposed to industrial noise. As a result, the authors determined the relationship between exposure to industrial noise and qualitative and quantitative morphological changes in rats' teeth, recognizing industrial noise as an aggressive stimulus for the pulp-dentin complex.

The fourth published original research article by Karamehmedovic et al. discussed the oral health situation in the Balkan area of Europe. The authors stated that the oral health situation for children in the Balkan region is poor; therefore, the authors aimed to identify obstacles that prevent the implementation of an effective oral health program for children in the Balkan area. The study investigates awareness, possibility to attend dental care, and existing barriers related to oral health promotion and disease prevention in a region where information is deficient. The authors suggested that the obtained results can be used to promote and improve oral health for children in the Balkan area and even wider.

The fifth published original research article aimed to increase knowledge of the impact of X-linked hypophosphatemia (XLH) on oral health. XLH is a rare genetic disorder caused by a mutation in the phosphate regulating endopeptidase homolog X-linked gene, affecting the skeleton and dentition with hypophosphatemia, causing abnormal mineralization of bone and dentine, rickets, osteomalacia, and short stature. In this study Larsson et al. confirmed that patients with XLH had a significantly lower oral health status compared to a healthy population, especially concerning endodontic conditions and apical pathology of clinically intact teeth. Furthermore, male patients with XLH had a higher risk of poor oral health compared to female patients with XLH, highlighting the need for individual gender risk analyses during treatment.

We would like to extend our congratulations to the research partners (Bullock et al. Rattanawonsakul et al. Cavacas et al. Karamehmedovic et al. Larsson et al.) who shared their discoveries within this topic.

